# Spatial soil temperature measurement data around an underground power cable under controlled thermal loading

**DOI:** 10.1016/j.dib.2026.112582

**Published:** 2026-02-11

**Authors:** Shahbaz Ahmad, Zarghaam Haider Rizvi, Frank Wuttke

**Affiliations:** a University of Kiel, Germany; b Kiewit Corporation, Denver, CO, USA; c University of Waterloo, Ontario, Canada

**Keywords:** Underground power cables, Soil thermal response, Cyclic thermal loading, Energy geotechnics, Large-scale laboratory experiment, Temperature time-series data

## Abstract

This article presents a large-scale experimental dataset describing the spatially and temporally resolved soil temperature field around a cable-representative heat source embedded in dry sand. A cylindrical electrical heater was used to represent an underground power cable conductor and operated under controlled steady-state and cyclic thermal loading conditions. The dataset includes long-duration steady-state heating at two target temperatures (70 °C and 90 °C), as well as symmetric and asymmetric cyclic heating–cooling regimes at 90 °C. Temperature time series were recorded using thermocouples installed at radial distances of 5, 10, 15, and 20 cm from the heat source, with sensors positioned above, beside, and below the heater to capture vertical temperature gradients. Additional measurements include heater surface temperature and ambient laboratory temperature. The present article consolidates the complete time-resolved dataset and associated metadata to support reuse, benchmarking, and further investigation of soil thermal behavior under controlled thermal loading conditions.

## Highlights

• Large-scale experimental dataset of soil temperature measurements around a cable-representative heat source under controlled thermal loading.
• Time-resolved temperature data recorded at multiple radial and vertical locations for steady-state and cyclic heating regimes.
• Dataset includes long-duration steady-state heating and symmetric and asymmetric cyclic heating–cooling conditions.
• Publicly available dataset suitable for benchmarking, model validation, and data-driven analysis of soil thermal behavior.

Specifications Table

**Table:** 

Subject	Engineering
Specific subject area	Energy geotechnics; thermal behavior of soils around underground power cables
Type of data	Time-series temperature data
Data format	Raw
How data were acquired	Temperature measurements using thermocouples installed at fixed radial and vertical locations around a temperature-controlled cylindrical electrical heater embedded in dry sand
Data collection	Large-scale laboratory experiments under steady-state and cyclic thermal loading
Data source location	Large-scale soil testing facility, Institute of Geosciences, University of Kiel, Kiel, Germany
Data accessibility	AHMAD, SHAHBAZ (2026), “Spatial soil temperature measurements around a power cable.”, https://opendata.uni-kiel.de/receive/fdr_mods_00000330
Related research article	Ahmad et al., *Scientific Reports* (2025)

## 1 Value of the Data

• The dataset provides large-scale, time-resolved measurements of soil temperature response under controlled thermal loading representative of underground power cable operation.
• The data can be used by cable designers, practicing engineers, and infrastructure professionals for cable route analysis, thermal backfill assessment, and design-oriented evaluation of underground power cable systems.
• The dataset enables benchmarking and validation of analytical, numerical, and empirical models used for cable ampacity calculations and soil heat transfer analysis.
• The inclusion of multiple steady-state and cyclic thermal loading regimes allows comparative assessment of long-term and transient heating effects on soil temperature fields.
• The spatially resolved sensor configuration supports investigation of radial and vertical thermal gradients around a buried heat source and facilitates the development and calibration of large-scale numerical and physics-based simulation models.
• The dataset is suitable for data-driven modeling approaches, including machine-learning applications involving transient soil thermal behavior.
• The dataset supports practical verification of underground power cable thermal rating assumptions by providing long-duration steady-state and cyclic temperature data consistent with IEC- and IEEE-based design and operational conditions.

## 2 Background

Underground power cables are a critical component of modern electrical infrastructure, and their thermal performance directly influences current-carrying capacity, operational reliability, and service life. Heat generated within a buried cable must be dissipated through the surrounding soil, making soil thermal behavior a governing factor in cable design, route selection, and rating calculations [[1], [2], [3]].

Previous studies have investigated the thermal behavior of underground power cables under steady-state and cyclic loading conditions, including the effects of dry-zone formation, temperature-dependent soil properties, and artificial backfill materials using analytical, numerical, and rating-based approaches [[2], [3], [4]]. Accurate representation of this behavior requires experimental data that capture both the spatial distribution and temporal evolution of soil temperature under controlled thermal loading conditions representative of practical cable operation.

In engineering practice, steady-state thermal loading conditions are commonly adopted for underground cable design and rating, reflecting sustained operating currents and long-term thermal equilibrium assumptions embedded in international standards [[5], [6], [7], [8]]. Analytical formulations such as those provided in IEC 60,287 and corresponding IEEE cable rating guidelines rely on steady-state representations to define allowable ampacity and conductor temperature limits. However, underground power cables in service are frequently subjected to time-varying loading arising from fluctuations in demand, operational scheduling, and network constraints. These conditions result in cyclic heating and cooling of the cable–soil system, producing transient thermal responses that are not fully captured by steady-state assumptions alone. Recent data-driven investigations of infrastructure embedded in soil environments [9] underscore the value of experimental datasets for capturing soil-controlled response mechanisms.

Cyclic thermal loading may occur under symmetric or asymmetric heating–cooling patterns, depending on the duration and sequence of load variations. Symmetric cycles, characterized by comparable heating and cooling periods, are representative of regular load modulation [[10], [11], [12]], while asymmetric cycles reflect conditions in which heating and cooling durations differ, leading to delayed thermal relaxation in the surrounding soil. Short-duration heating–cooling cycles provide time-resolved information on rapid thermal transients, whereas longer cooling intervals capture the persistence of heat stored within the soil. Experimental datasets that systematically document soil temperature behavior under steady-state, symmetric cyclic, and asymmetric cyclic thermal loading remain limited, particularly at laboratory scales representative of underground cable installations.The selection of heating temperature levels in experimental investigations is guided by standard-defined operating limits for underground power cables. A conductor temperature of approximately 90 °C is widely recognized as the maximum continuous operating temperature for cross-linked polyethylene insulated power cables under normal service conditions, as specified in IEC and IEEE provisions for cable design and operation. Lower conductor temperatures, such as 70 °C, are commonly associated with conservative design assumptions, partial loading conditions, or prolonged operation below the maximum allowable thermal limit. Inclusion of both temperature levels enables the dataset to capture soil thermal response under moderate and near-limit operating conditions that are directly relevant to engineering practice.

Although analytical models and numerical simulations are widely used to evaluate steady-state and transient thermal behavior in underground cable systems, their reliability depends strongly on the availability of high-quality experimental data for calibration, validation, and benchmarking. Existing experimental studies often focus on a limited subset of loading scenarios or present only processed results, constraining their applicability for comparative assessment and large-scale model development. To address this gap, a large-scale laboratory experimental program was conducted to measure soil temperature fields around a cable-representative heat source embedded in dry sand under controlled thermal loading conditions. The testing program incorporated long-duration steady-state heating as well as symmetric and asymmetric cyclic heating–cooling regimes at target temperatures aligned with IEC and IEEE operating provisions, resulting in a consolidated dataset relevant to underground power cable design, cable route analysis, and thermal modeling.

## 3 Data Description

The dataset comprises time-resolved temperature measurements collected during a large-scale laboratory experimental program investigating soil thermal behavior around a cable-representative heat source embedded in dry sand. The data are organized according to thermal loading regime and operating temperature, and they include raw temperature time series together with sensor location metadata and experimental condition descriptors.

In practical engineering applications, reliable thermal rating of underground power cables requires experimental data that reflect realistic operating temperatures, loading durations, and spatial heat dissipation patterns. The experimental program underlying this dataset was therefore designed to align with IEC and IEEE operating provisions by incorporating extended steady-state heating and controlled cyclic loading at conductor-representative temperature levels. The resulting dataset provides application-oriented temperature measurements that can be directly used for thermal rating verification, calibration of numerical models, and assessment of transient thermal behavior under operational cable loading scenarios.

### 3.1 Dataset overview

The dataset includes temperature measurements recorded under the following controlled thermal loading conditions:

• Steady-state heating at 70 #x00B0;C
  Continuous heating applied for a total duration of 120 h.
• Steady-state heating at 90 #x00B0;C
  Continuous heating applied for a total duration of 335 h.
• Symmetric cyclic heating at 90 #x00B0;C
  Heating–cooling cycles consisting of 1 h heating followed by 1 h cooling, with a total test duration of 9 h.
• Asymmetric cyclic heating at 90 #x00B0;C
  Heating–cooling cycles consisting of 1 h heating followed by 2 h cooling, with a total test duration of 9 h.

For all test conditions, temperature data were recorded continuously at fixed spatial locations relative to the heat source. The durations of the steady-state heating tests were selected to ensure that the soil temperature field within the instrumented zone approached thermal equilibrium. Steady-state conditions were verified by monitoring the temporal evolution of temperature at all sensor locations and confirming that temperature variations became negligible over extended periods. The longer steady-state duration applied at 90 °C reflects the higher imposed temperature gradient and thermal inertia associated with near-limit operating conditions relevant to underground power cable design.

### 3.2 Sensor configuration and measurement locations

Temperature measurements were obtained using thermocouples installed within the soil at multiple radial and vertical positions relative to the cable-representative heat source. The spatial configuration was consistent across all test scenarios.

• Radial distances from the heat source surface: 5 cm, 10 cm, 15 cm, and 20 cm
• Vertical positions at each radial distance:
• Above the heat source
• Beside the heat source
• Below the heat source

This configuration resulted in three thermocouples at each radial distance, enabling characterization of vertical temperature gradients within the soil. In addition to the soil-embedded sensors, dedicated thermocouples were installed to monitor heater surface temperature and ambient laboratory conditions. See table 1 for description of the thermocouples.

**Table 1:** Description of the thermocouples.

Position of the thermocouples	Name and respective distances of the thermocouples*
Heater surface⁎⁎	A24, A10, A30, A31
Outside container	A0 (86), A8 (40)
Above heater	A7 (5), A11 (10), A14 (15), A26 (20), A9 (25), A27 (30)
Below heater	A20 (5), A4 (10), A2 (15), A1 (20), A3 (25), A19 (30), A15 (40), A12 (50), A25 (70)
Horizontal direction	A18 (5), A21 (10), A17 (15), A5 (20), A16 (25), A6 (30), A28 (40), A22 (50), A23 (55), A13 (85)

⁎ Distances are in cm from the surface of the heater rod, with the distance mentioned in parentheses.

⁎⁎ The reported heater temperature corresponds to the centrally located heater sensor, used as the reference control temperature in all tests.

During each experimental test case, temperature measurements were recorded simultaneously from all installed thermocouples at multiple radial and vertical positions relative to the heater. The study identifiers listed in Table 2 correspond to specific thermal loading conditions. Table 3 presents the subset of thermocouples selected for each study type to illustrate soil temperature response at specific radial distances from the heat source. These subsets include thermocouples located above, beside, and below the heater at the corresponding radial distance, allowing focused assessment of radial and vertical temperature gradients. The complete dataset, including all thermocouple channels, is provided in the deposited data files.

**Table 2:** Summary of experimental test cases and associated thermal loading conditions.

Data File Name	Corresponding Study Type	Study Acronym	Duration (hours)
SS70_5cm.xlsx	For Steady State Heating Conditions of the Heater operated at 70 Deg C (focus on radial distance of 50 mm from heater surface)	SS-1	120
SS70_Dry10.xlsx	For Steady State Heating Conditions of the Heater operated at 70 Deg C (focus on radial distance of 150 mm from heater surface)	SS-2	120
SS70_Dry15.xlsx	For Steady State Heating Conditions of the Heater operated at 70 Deg C (focus on radial distance of 100 mm from heater surface)	SS-3	120
SS70_Dry20.xlsx	For Steady State Heating Conditions of the Heater operated at 70 Deg C (focus on radial distance of 200 mm from heater surface)	SS-4	120
90SS_5.xlsx	For Steady State Heating Conditions of the Heater operated at 90 Deg C (focus on radial distance of 50 mm from heater surface)	SS-5	335
90SS_10.xlsx	For Steady State Heating Conditions of the Heater operated at 90 Deg C (focus on radial distance of 150 mm from heater surface)	SS-6	335
90SS_15.xlsx	For Steady State Heating Conditions of the Heater operated at 90 Deg C (focus on radial distance of 100 mm from heater surface)	SS-7	335
90SS_20.xlsx	For Steady State Heating Conditions of the Heater operated at 90 Deg C (focus on radial distance of 150 mm from heater surface)	SS-8	335
1hrdata.xlsx	For Cyclic Heating Conditions of the Heater operated at 90 Deg C (focus on radial distance of 50 mm from heater surface) with 1 hour heating and 1 hour cooling.	Sym	9
2hrdata.xlsx	For Cyclic Heating Conditions of the Heater operated at 90 Deg C (focus on radial distance of 50 mm from heater surface)with 1 hour heating and 2-hour cooling.	Unsym	9

**Table 3:** Description of the data in the excel sheets arranged as per the thermocouple name.

Study	Column A	Column B	Column C	Column D	Column E	Column F	Column G
SS-1	Duration (hours)	A7	A18	A20	A0	A8	Heater
SS-2	Duration (hours)	A11	A21	A4	A0	A8	Heater
SS-3	Duration (hours)	A14	A17	A2	A0	A8	Heater
SS-4	Duration (hours)	A26	A5	A1	A0	A8	Heater
SS-5	Duration (hours)	A7	A18	A20	A0	Heater	-
SS-6	Duration (hours)	A11	A21	A4	A0	Heater	-
SS-7	Duration (hours)	A14	A17	A2	A0	Heater	-
SS-8	Duration (hours)	A26	A5	A1	A0	Heater	-
Sym	Complete data in file name 1 hr data. Refer to the table of thermocouples for details regarding the distance.						
Unsym	Complete data in file name 2 hr data. Refer to the table of thermocouples for details regarding the distance.						

### 3.3 Data content and format

The dataset consists of raw temperature time series recorded at regular sampling intervals throughout each test. Each data file (excel or .csv file) contains:

• Time stamps
• Temperature measurements for each thermocouple are in degree C.
• No filtering, smoothing, or post-processing has been applied to the temperature measurements. All data are provided in raw form to allow independent analysis and reuse.

### 3.4 Data reuse considerations and observations

The dataset is suitable for reuse in applications including cable route analysis, underground cable design, benchmarking of analytical and numerical heat transfer models, and development of large-scale or data-driven thermal simulations. The consistent sensor layout and inclusion of multiple thermal loading regimes enable comparative assessment of soil temperature behavior under steady-state and transient operating conditions. Based on the experimental dataset presented in this article, the following key observations and considerations are highlighted to support appropriate data interpretation and reuse:

• The dataset captures both steady-state and cyclic thermal responses of soil around a cable-representative heat source, enabling comparison between stabilized and transient thermal regimes under controlled laboratory conditions.
• Temperature measurements at multiple radial and vertical locations reveal non-uniform spatial temperature distributions, including higher temperatures above the heater compared to below, reflecting asymmetric boundary conditions and three-dimensional heat dissipation.
• Cyclic thermal loading data preserve complete heating–cooling sequences at high temporal resolution, allowing users to examine transient thermal oscillations, phase lag, and cumulative thermal effects without imposed analytical assumptions.
• The experimental conditions were intentionally selected to isolate conduction-dominated heat transfer in dry sand, providing a reproducible reference dataset suitable for benchmarking analytical solutions and validating numerical models.

These observations are intended to assist researchers and practitioners in effectively utilizing the dataset for independent analysis, model development, and comparative studies.

### 3.5 Representation of steady-state and cyclic thermal loading in the dataset

The experimental program was designed to capture both stabilized and transient soil temperature responses around a cable-representative heat source under controlled laboratory conditions. Steady-state thermal loading was implemented by maintaining constant heater surface temperatures of 70 °C and 90 °C, selected to represent typical maximum operating limits of underground power cables. Temperature measurements were recorded at radial distances ranging from 5 to 20 cm from the heater, with thermocouples positioned above, beside, and below the heat source to resolve both radial and vertical temperature gradients.

During steady-state heating, soil temperatures increased progressively and reached quasi-equilibrium conditions after extended heating durations, beyond which no significant temporal variations were observed. The recorded data consistently show higher temperatures in the soil above the heater compared to below, reflecting the asymmetric placement of the heater within the sandbox and the reduced soil cover above the heat source. This configuration promotes enhanced upward heat transfer and results in clear vertical temperature gradients, which are captured across all monitored radial distances.

Cyclic thermal loading was applied to represent time-varying operating conditions through prescribed heating–cooling sequences at the same heater surface temperatures. Two cyclic loading patterns were implemented: a symmetric cycle consisting of 1 h heating followed by 1 h cooling, and an asymmetric cycle consisting of 1 h heating followed by 2 h cooling. The temperature time-series data record periodic thermal oscillations synchronized with these cycles at all monitored radial and vertical locations.

In addition to the cyclic oscillations, the data exhibit a gradual upward shift in baseline soil temperature over successive cycles, indicating cumulative thermal charging of the surrounding soil. This effect is most pronounced in the vicinity of the heater but remains observable at larger radial distances. Similar to the steady-state tests, higher temperatures are consistently recorded above the heater than below during cyclic loading, indicating preferential upward heat propagation and vertical channelization of heat under transient thermal conditions.

Together, the steady-state and cyclic datasets provide complementary representations of soil thermal behavior, capturing stabilized temperature distributions, transient oscillations, vertical temperature asymmetry, and cumulative thermal effects. The dataset enables users to examine spatial and temporal temperature response characteristics directly from the recorded measurements, without imposing analytical assumptions or model-specific interpretations.

## 4 Experimental Design, Materials and Methods

### 4.1 Experimental setup

**Fig. 1:**
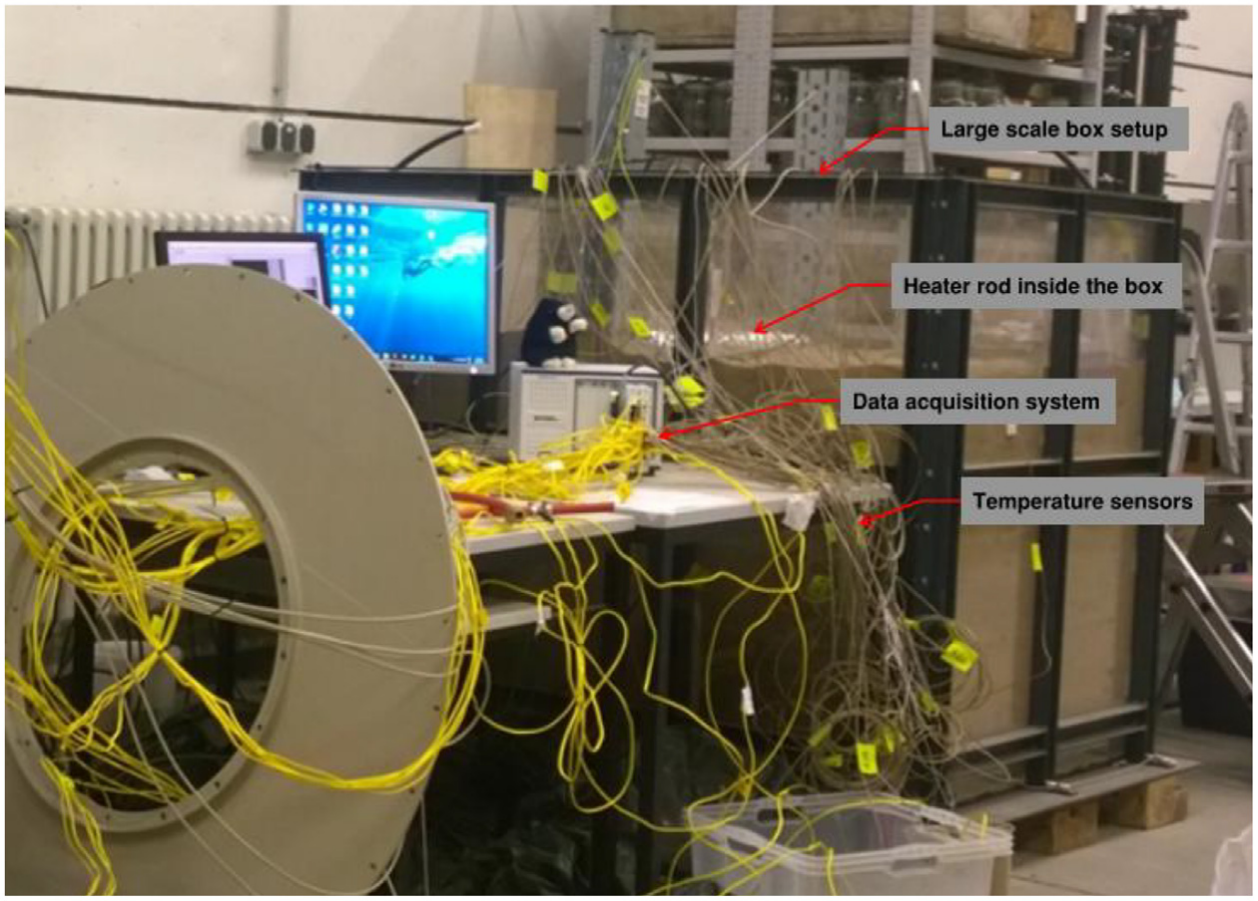
Large-scale laboratory experimental setup used for temperature data acquisition, illustrating the instrumented soil box, thermocouples cabling network, and data acquisition system.

**Fig. 2:**
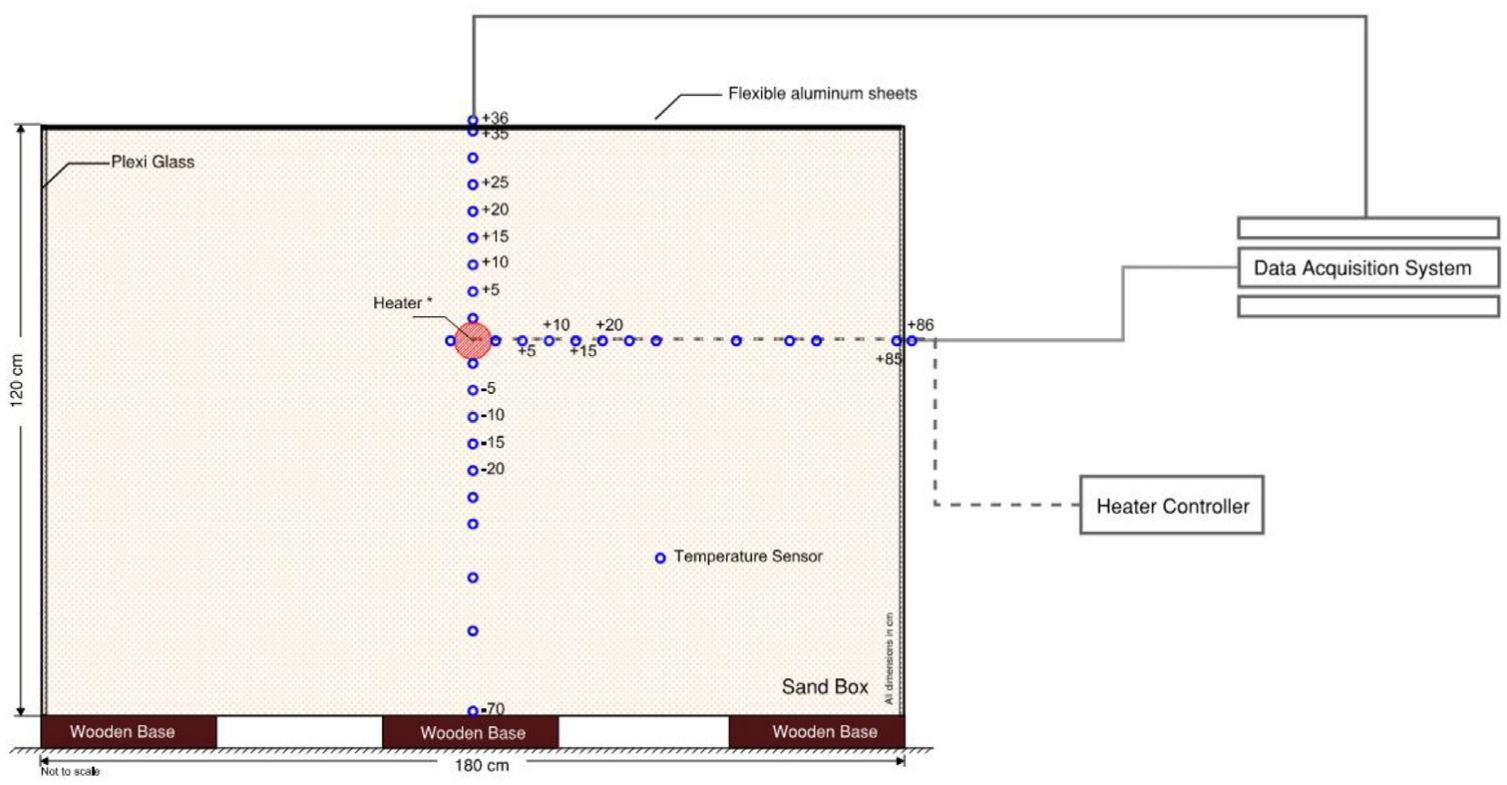
Schematic diagram of experimental setup with heating control unit and thermocouple arrangement with data acquisition unit.

The experimental program was conducted using a large-scale laboratory apparatus designed to simulate an underground power cable trench under controlled thermal loading conditions, shown in Figs. 1–2. The setup consisted of a rectangular sandbox supported by a heavy-duty steel frame to safely accommodate the soil mass and testing equipment. The sandbox had internal dimensions of 1800 mm (length) × 1000 mm (width) × 1200 mm (height), see Fig. 2.

The side walls of the sandbox were constructed using Plexiglas panels to allow visual observation of the soil and sensor placement during preparation and testing. Plexiglas was selected instead of conventional glass due to its higher resistance to thermal shock and mechanical damage. Rubber linings were installed along the panel edges to ensure water-tight conditions and to enhance operational safety. The top surface of the sandbox was covered with flexible aluminum sheets to limit heat loss to the surrounding laboratory environment. To further reduce conductive heat transfer, the sandbox was isolated from the laboratory floor using rubber sheets and solid wooden planks.

A cable-representative heat source was implemented using a tubular electrical heating rod with a length of 800 mm and a diameter of 50 mm. The heating rod was installed horizontally and oriented parallel to the 1000 mm side of the sandbox. It was positioned at a vertical distance of 750 mm from the bottom of the container and held rigidly between two vertical supports to maintain stable alignment throughout the testing program.

The experimental scale and heater placement were selected to minimize boundary effects on the measured temperature field. All soil temperature sensors were installed within a maximum radial distance of 200 mm from the heater surface, ensuring that the instrumented zone was well separated from the container boundaries in all directions. This configuration ensured that the observed temperature evolution within the measurement domain was governed primarily by heat transfer within the soil mass rather than by external boundary constraints, including during long-duration steady-state heating tests of up to 335 h.

To minimize potential thermal shortcuts and non-radial heat transfer, the sandbox was physically isolated from the laboratory floor using rubber sheets and solid wooden planks, thereby reducing conductive heat loss through the base. The container walls were constructed from Plexiglas panels, which limited lateral heat transfer while allowing visual inspection of the soil and sensor placement. The top surface of the sandbox was covered with flexible aluminum sheets to reduce convective and radiative heat exchange with the surrounding environment.

Within the instrumented zone, all thermocouples were located at distances significantly smaller than the separation between the heater and the container boundaries. As a result, heat transfer within the measurement region was governed primarily by soil conduction, and the assumption of quasi-radial heat flow around the heating rod is considered valid for the purposes of the reported dataset.

The described support and insulation measures ensure that the reported temperature data represent soil-dominated heat transfer behavior suitable for use as a benchmark dataset in analytical and numerical thermal modeling studies.

### 4.2 Soil material preparation

Dry sand was used as the backfill material for all tests to provide a homogeneous and repeatable soil medium. Prior to placement, the sand was dried to remove moisture and then placed in layers within the container to achieve uniform density. Geotechnical laboratory tests indicate that the sand is classified as uniform sand, with a porosity of 0.36. The thermal conductivity measurements were performed with Decagon KD2 Pro device. The measured thermal conductivity values was 0.365 W/m K for dry sand.

Care was taken during placement to avoid segregation and to ensure consistent thermal contact between the soil and the embedded sensors.

The use of dry sand allowed isolation of conductive heat transfer mechanisms and reduced variability associated with moisture migration, enabling controlled investigation of soil temperature response under different thermal loading regimes.

### 4.3 Heat source and thermal loading control

The cable-representative heat source consisted of a cylindrical electrical heater operated in temperature-controlled mode. Heater surface temperature was monitored using a dedicated thermocouple attached directly to the heater surface and regulated through a control system to maintain target temperature levels.

The experimental program included both steady-state and cyclic thermal loading conditions. For steady-state tests, the heater was operated continuously at target surface temperatures of 70 °C and 90 °C for durations of up to 120 h to allow the surrounding soil to approach thermal equilibrium. For cyclic tests, the heater was operated at a target temperature of 90 °C using predefined heating–cooling schedules, including symmetric cycles with equal heating and cooling durations and asymmetric cycles with longer cooling periods. The total duration of each cyclic test was 9 h.

The temperature controller provided real-time indications of electrical power required to maintain the prescribed heater surface temperature; however, these values were not logged or averaged over time. Indicative power levels on the order of ∼83 W/m were observed during operation but are not reported as quantitative power inputs within the dataset.

### 4.4 Temperature measurement and instrumentation

Soil temperature measurements were obtained using thermocouples installed at fixed spatial locations relative to the heat source. Sensors were positioned at radial distances of 5 cm, 10 cm, 15 cm, and 20 cm from the heater surface. At each radial distance, three thermocouples were installed to capture vertical temperature variation, with sensors positioned above, besides, and below the heat source.

In addition to the soil-embedded thermocouples, one thermocouple was attached to the heater surface to monitor the imposed thermal boundary condition, and one thermocouple was used to record ambient laboratory temperature throughout the tests. All thermocouples were connected to a data acquisition system that recorded temperature measurements at regular sampling intervals during each test.

### 4.5 Data acquisition and quality control

Temperature data were recorded continuously throughout each test using a multi-channel data acquisition system. Time stamps were synchronized across all channels to ensure consistent temporal alignment of measurements. No filtering, smoothing, or averaging was applied during data collection; all measurements were stored in raw form.

Basic quality control checks were performed to verify sensor functionality and continuity of data recording. Tests were conducted under controlled laboratory conditions to minimize external thermal disturbances. Ambient temperature was monitored to document boundary conditions during the experiments.

### 4.6 Reproducibility and dataset scope

The experimental configuration, sensor layout, and thermal loading protocols were kept consistent across all test scenarios to enable direct comparison between steady-state and cyclic heating conditions. The dataset represents the complete set of temperature measurements generated during the experimental program and is provided together with accompanying metadata describing sensor locations, test durations, and loading regimes.

### 4.7 Instrumentation details

Temperature measurements were obtained using National Instruments K-type thermocouples installed at predefined radial and vertical locations relative to the heating rod. In total, thirty-two thermocouples were used to monitor soil and heater temperatures, while two additional thermocouples were placed outside the testing apparatus to record ambient temperature. The K-type thermocouples have an operating range of −55 °C to 550 °C with a manufacturer-reported accuracy of ±0.5 K. Independent cross-calibration against Pt-100 resistance temperature detectors showed deviations smaller than ±0.3 K, confirming reliable temperature measurement performance.

Heater surface temperature was monitored using four thermocouples positioned circumferentially at the top, bottom, and opposing sides of the heating rod, and the average of these measurements was used to represent the heater temperature. All thermocouples were connected to a multichannel data acquisition system, and temperature data were recorded at a uniform sampling interval of 10 s for all experimental test cases, including steady-state and cyclic thermal loading. This sampling interval was selected to adequately resolve transient temperature variations during the 1-hour cyclic loading tests while ensuring stable long-term data recording.

## Limitations

The dataset was generated under controlled laboratory conditions using dry sand to provide a homogeneous and repeatable test medium and to isolate conductive heat transfer. Consequently, the data does not capture soil moisture effects, which may be present in field conditions. The cable-representative heat source simulates conductor heating but does not include insulation layers, metallic sheaths, or installation components. In addition, the experiments were conducted under uniform boundary conditions and horizontal installation geometry, and do not account for site-specific heterogeneity or environmental influences. These factors should be considered when applying the dataset to field-scale scenarios.

## Ethics Statement

This study did not involve human participants, animals, or personal data. No ethical approval was required for the conduct of the experiments or for the publication of the dataset.

## CRediT Author Statement

**Shahbaz Ahmad:** Conceptualization, Data curation, Formal analysis, Investigation, Methodology, Project administration, Validation, Visualization, Writing – original draft, Writing – review & editing. **Zarghaam Rizvi:** Conceptualization, Data curation, Formal analysis, Funding acquisition, Investigation, Methodology, Project administration, Supervision, Validation, Visualization, Writing – original draft, Writing – review & editing. **Frank Wuttke:** Conceptualization, Funding acquisition, Investigation, Methodology, Project administration, Resources, Software, Supervision, Writing – review & editing.

## Data Availability

The data set described in this article is publicly available through the University of Kiel Open Data repository. AHMAD, SHAHBAZ (2026), “Spatial soil temperature measurements around a power cable.”, Spatial soil temperature measurements (Original data)https://opendata.uni-kiel.de/receive/fdr_mods_00000330.
